# Information Regarding the Stereopticon Loan Library of the U. S. Public Health Service

**Published:** 1916-10

**Authors:** 


### Information Regarding the Stereopticon Loan Library of the U. S. Public Health Service.

The stereopticon loan library established by the United States Public Health Service consists of over 2000 views, the majority of which are original, dealing with the aspects of various public health problems. Additions are constantly being made to the collection. The slides are classified by diseases or subjects, the following being the respective divisions of the library:

*Alaska.*—Eighty-three views depicting living conditions in the territory of Alaska.

*Children and Children's Diseases.*—The various eruptive diseases of children are shown in 50 views. Chiefly of interest to physicians.

*Hookworm.*—The geographic distribution of the disease, its economic importance, the life history of the parasite, its invasion of human tissue and the resulting effects are demonstrated in a series of over 90 slides.

*Indians.*—Housing and living conditions among American Indians. Shown in 50 views.

*Leprosy.*—Forty-five slides depicting the disease. Principally of service to physicians.

*Living Conditions.*—Contains a relatively small number of slides. See other subjects.

*Malaria.*—Prevalence of the disease, the malarial parasites, larval, pupal and adult developmental stages of mosquitoes, breeding places, methods of extermination, including oiling, drainage and the types of fish destructive to larvae. Prevention of the disease by screening and the use of quinine. Two hundred and seventy-five views.

*Milk.*—Eighty views showing tuberculous cows, proper and improper stabling, care and treatment of dairy herds, methods of obtaining pure milk, spread of milk-borne epidemics and the value of sanitary measures.

*Miscellaneous Subjects.*—Sewage disposal, fumigation and cleaning of railway cars, and views relating to Rocky Mountain spotted fever.

*Mouth Hygiene.*—Twelve slides showing the development of the teeth.

*Parasites and Organisms.*—Over two hundred views of the common organisms causing the diseases of man, including different types of water organisms. Also the developmental stages of fleas, lice, flies, and disease-bearing vermin.

*Pellagra.*—Statistical data, geographical distribution and the lesions of the disease presented by 60 photographic slides.

*Plague.*—Perhaps the most complete collection of original plague slides extant. Over five hundred views.

*Rural Schools.*—Not yet complete. Ten slides.

*Smallpox.*—Ninety slides illustrating the eruptive stages of the disease, the protection afforded by vaccination and the lesions thereof.

*Trachoma.*—The disease in its acute and chronic stages, and such effects as pannus, entropion and blindness.

*Tropical Diseases.*—Common infections of the tropics.

*Tuberculosis.*—One hundred slides showing the economic loss from tuberculosis, susceptible races, the tubercle bacillus, pathological conditions in the lungs, the relation of the disease to improper housing and the causes predisposing to infection.

*Typhoid Fever.*—Three hundred and fifty views.

*Yellow Fever.*—Mosquitoes in different stages of development, preventive measures, including detention camps. The discoverers of the means of transmission of the disease.

---

### HOW TO USE THE STEREOPTICON LOAN LIBRARY.

The slides are loaned to physicians, health organizations, educators, welfare workers, and others, without cost. Persons desiring slides should advise the Bureau as to what subjects they are interested in, so that the proper catalogues may be forwarded. The slides should be selected by number, and the request made upon the application blank. If desired, the Public Health Service will undertake to make the selection, provided the applicant will state what he wishes to illustrate. There is no arbitrary limit within which the slides are to be returned, but as the demand far exceeds the supply it is expected that they will be returned at the earliest possible moment. Stereopticon lanterns are not loaned, but as the slides are of standard size, 3¼ by 4 inches, any lantern may be used. It is expected that slides broken by careless handling or packing will be replaced; these to be ordered from the government contractor by the United States Public Health Service and the bill therefor to be paid by the borrower.

It is requested that in returning the slides a letter of transmittal be forwarded, stating the approximate number of persons to whom the views have been shown. The container should be labeled with the name and address of the sender, and returned by express prepaid or by mail. Photographs, from which it is possible to obtain slides of public health interest, will be gladly received and promptly returned.

